# Gum Boils—Fungous Growth of the Gums.—Epulis

**Published:** 1849-07

**Authors:** 


### GUM BOILS—FUNGOUS GROWTH OF THE GUMS.—EPULIS.

I have already spoken of the bad effects of decayed teeth and stumps, and have told you that an abscess occasionally forms at the fangs of the teeth. You will find now and then, on taking out the stump of a tooth, that there is a swelling, and on examination, you discover that it is a cyst, containing puriform matter. Sometimes these cysts are of very large size. I have seen a cyst—a complete abscess—as large as the tip of the finger, come away on removing a tooth. These abscesses even break externally, and if any of the cyst is left, matter will continue to be discharged for some time. These abscesses sometimes so increase in size, as to lead to a swelling of the jaws. An abscess formed at the socket of the tooth, now and then makes its way along the tooth. If the sockets have been a great deal absorbed, the matter at last comes up, and presents itself under the gum. It is described under the name *parulis* —gum-boil. There is a superficial swelling of the gum, followed by suppuration; but the troublesome cases are those in which abscesses form in the very sockets of the teeth; they are attended with great pain, swelling of the face, and so on. The abscess gradually advances, and may be discovered fluctuating very distinctly. You open the mouth and see a large swelling on the upper or lower jaw, which you find to be elastic, and on putting a lancet into it, there is a great escape of putrid matter, which is attended with much relief to the patient. If a patient has suffered from this once, the cause ought to be explained, and means ought to be taken to prevent the recurrence of it. He is unwilling to have the teeth taken out when the parts are quiet and going on well, but some of these collections form deep in the jaws; they go on increasing, the parieties of the abscess expand, and cavities in the bone are at last formed of considerable size. When formed in the upper jaw, the abscess may burst into the antrum, and sometimes there is a cavity, independently of that in the upper jaw, a large chronic abscess. The same thing occurs in the lower jaws. Sometimes the plates of the bone separate to a great extent, and if neglected for some time, you find tumors formed, of very large size, which are gradual and slow in their progress. These cases are known under the name of *spina ventosa.* It is only in patients who have been neglected, and in whom the disease has been allowed to increase from month to month, that anything of this kind is observed.

Then, again, in removing decayed teeth, or portions of them, which have been allowed to remain long, you will now and then perceive a fungous growth on the extremity—a soft, pulpy swelling, adherent to the apex of the fang. Again: if you notice a carious tooth when extracted, you will sometimes find a soft fungus in the hollow of it, and if you take the trouble of the splitting it up with a pair of cutting pliers, you will find that the whole canal is filled up by a swelling, which expands like a mushroom. These swellings often increase in size; they fill up the remainder of the crown of the tooth, and sometimes form a connection with the spongy gums. In other cases, the swelling commences at the gum, by the side of the decayed tooth; it gradually increases in size, and perhaps involves the gums of the adjoining teeth. Some of these swellings are as hard as the gum; some are soft and pulpy, and bleed on a slight touch; and some again, though very seldom, assume a malignant character. These tumors are generally of a benign nature, they are firm in their consistence, and, if thoroughly extirpated, are not reproduced, but if any portion is left, they return. If the socket of the tooth, in which the disease commenced, is not taken away, and indeed the whole gum, the disease is sure to come back in a few months, following the analogy of tumors in other parts. I have told you that, however benign in its nature a fibrous or fatty tumor may be, if any portion of it be left, it will be reproduced; but take away the whole, and there is little chance of the patient being again troubled with it.

These tumors sometimes are of a bad character; but even in those of a contrary nature, where the operation is imperfectly performed, there is a return of the disease. The patient much annoyed of course, again recurs to his surgeon; caustic is perhaps applied from day to day; being alarmed, he at last places himself under a person of more experience; the whole is then taken away, and there is no further trouble. Here are some drawings [exhibited] from preparations belonging to Mr. Nasmyth, of Edinburg, showing tumors of the gums. One represents a tumor occupying the posterior part of the upper jaw, with all the stumps stuck in the middle of it. The teeth are all in a bad state. Those persons who are foolish enough to allow useless portions of the teeth to remain, may lay their account to suffer from this disease. The pain has gone off, the nerve is destroyed, and they think there is no occasion for interfering with the teeth, or having them taken out. They do not care about the fœtor of their breath; they have perhaps arrived at a time when they think nothing of it; but there is always a deal of mischief if these stumps in the jaws or gums are not taken out. They keep up the swelling and the tumors in the gums. Here is another drawing, [exhibited] showing a tumor of the gum, where the swelling has gone up from the interior of the tooth, and has spread over it in a mushroom-like form, and becoming adherent to the spongy gums, has formed a large swelling. The following is an extract from a letter which I lately received from Mr. N.:—

"I was much interested in a notice of a clinical lecture of yours in the "Lancet" of Feb. 18th, and as I have some casts and preparations of cases which have had their origin very distinctly in carious teeth, I thought you would like to have some notice of them. You have often, I doubt not, observed a fungous growth sprouting up in the hollow of a carious tooth. This often goes on till it fills a large cavity, or rather the whole excavated crown of the tooth." My attention was drawn to this fact years ago. "By and by, the caries advancing, the attenuated walls give way, the growth in the centre of the tooth forms a connection with the gum, and then increases with rapidity. In the case No. 1, the tumor was connected with its base by a narrow neck; in No. 2, the base was broader, as you will observe, had spread the roots very much asunder.

"The principle value of these cases is of course to show that it is best in general to get rid of broken teeth, especially where there is a tendency to the fungous growth, or a relaxed spongy state of the gums."

From what I have observed, I have no doubt that he is correct in saying that these tumors of the gums, these cases of epuli, often depend upon a growth in the interior of the tooth, as well as in the gums.

In order to get rid of these tumors effectually, you must take away the whole of the growth. Most frequently you find them connected with the decayed fangs of the small grinders upon one side or the other of the jaw, and most frequently the lower jaw. Sometimes you find them far back in the lower jaw, growing from the decayed roots of the last large grinder, and spreading their influence to the gums of the wisdom-tooth and the grinder anterior to it. It is then a difficult matter to get quit of the swelling. If it be of large size, the patient can only open his mouth with difficulty, and you get but an imperfect view of it. In the fore part of the mouth there is no difficulty at all. All you have to do, then, is to extract a tooth, sound or unsound, on each side of the tumor; the gums are more or less involved in the disease, and you can take them out with the forceps. If the disease were connected with a canine tooth, you would then take out the first small molar tooth, and the lateral incisor; or suppose it were confined to the gums of the canine and first molar, then you would take away the lateral incisor of that side, and the second small molar. You then apply a small saw to the socket, and cut down the jaw, with a view of getting rid of the parts from which the disease has commenced, of removing the alveoli and the diseased sockets of the teeth. Before you apply the saw, you carry your knife round the base of the tumor, and having applied it, and cut down the bone on each side, then, by means of cross-cutting pliers, you remove the teeth and the sockets, together with the tumor. Here is a drawing [exhibited] representing a case in which the tumor had been taken away over and over again from the neck of the tooth, but it always grew again. The epulis was found adhering to the neck of the tooth, having commenced in the periosteum; but on that being removed there was no reproduction. When the gums of several teeth are affected, you must take away a tooth on each side, and cut away the sockets of all the teeth. If you do this, there is no necessity for any further proceeding, Some surgeons recommend that you should employ an escharotic, as the potassa fusa, to remove the disease more effectually, but this is unnecessary. It is better to go far enough with the saw and the forceps. In order to get rid of tumors far back, and to avoid the necessity of cutting open the cheek, it is necessary to have forceps of various sizes and forms. Although these instruments look very large and coarse, and such as one might say farriers would employ, yet they enable you to remove the affection with less trouble and pain to the patient, than if you use small and inefficient forceps. If you were to apply forceps half the size, you would find that you could not cut the sockets through cleverly; that they would bend, and you would have to repeat the operation; whereas, if you go properly to work, you will have no difficulty in accomplishing your object.—*Liston, Lecture IX*:—*Lancet.*

